# The saccadic Stroop effect: Evidence for involuntary programming of eye movements by linguistic cues

**DOI:** 10.1016/j.visres.2009.01.001

**Published:** 2009-03-30

**Authors:** Timothy L. Hodgson, Ben A. Parris, Nicola J. Gregory, Tracey Jarvis

**Affiliations:** a School of Psychology, University of Exeter, Prince of Wales Road, Devon EX4 4QG, UK; b Psychology Research Group, School of Design Engineering and Computing, University of Bournemouth, Poole, Dorset BH125BB, UK

**Keywords:** Oculomotor, Executive control, Response conflict, Automaticity, Language

## Abstract

The effect of automatic priming of behaviour by linguistic cues is well established. However, as yet these effects have not been directly demonstrated for eye movement responses. We investigated the effect of linguistic cues on eye movements using a modified version of the Stroop task in which a saccade was made to the location of a peripheral colour patch which matched the “ink” colour of a centrally presented word cue. The words were either colour words (“red”, “green”, “blue”, “yellow”) or location words (“up”, “down”, “left”, “right”). As in the original version of the Stroop task the identity of the word could be either congruent or incongruent with the response location. The results showed that oculomotor programming was influenced by word identity, even though the written word provided no task relevant information. Saccade latency was increased on incongruent trials and an increased frequency of error saccades was observed in the direction congruent with the word identity. The results argue against traditional distinctions between reflexive and voluntary programming of saccades and suggest that linguistic cues can also influence eye movement programming in an automatic manner.

## 1 Introduction

Unlike other animals humans can coordinate visuo-motor behaviour in response to spoken and written language. This process is usually assumed to rely on poorly specified cognitive mechanisms, although a number of studies have now shown how motor response programming can be invoked in a more “automatic” manner by linguistic and symbolic cue. These effects have been demonstrated using press button manual and vocal response reaction time measures (Hommel, Pratt, Colzato, & Godijn, 2001; Logan, 1995; Logan & Zbrodoff, 1998), although no research to date has directly investigated whether such effects occur in the control of eye movements.

A common task used to study such interactions in the non-oculomotor domain has been the Stroop task (MacLeod, 1991; Stroop, 1935). In this task, participants are asked to respond to the colour of the ink in which a word is printed, whilst ignoring the word itself. Sometimes the identity of the irrelevant word conflicts with the colour to be named (e.g. the word “red” printed in yellow). Increased response times and overt response errors are observed for such stimuli relative to those for which there is no conflict (e.g. “top” in yellow or “yellow” written in yellow). The influence of word identity cannot be entirely suppressed even when subjects are instructed to respond only to the colour in which it is printed.

Given the evidence for automatic activation of manual/vocal responses by linguistic cues in the Stroop task, it is notable that no studies have shown such effects on saccade programming**.** Instead, a large number of studies have demonstrated involuntary programming of saccades to peripheral visual onsets. For example, the latency of saccades increases under conditions for which a task irrelevant visual stimulus is presented prior to saccade execution (McSorley, Haggard, & Walker, 2004; Ross & Ross, 1980; Walker, Deubel, Schneider, & Findlay, 1997). The presence of “Distractor” stimuli also influences the spatial parameters of saccades (i.e. trajectory and amplitude) and often results in the execution of involuntary saccades (e.g. Hallett & Adams, 1980; Ludwig & Gilchrist, 2002; Theeuwes, Kramer, Hahn, & Irwin, 1998). The frontal lobe of the cerebral cortex is thought to mediate inhibitory influences over saccades in order to suppress the majority of such “capture errors” in healthy individuals. Damage in this region has been shown to be associated with increases in involuntary saccades to peripheral onsets (Hodgson, Chamberlain, Parris, James, Gutowski, Husain & Kennard, 2007; Guitton, Buchtel, & Douglas, 1985; Machado & Rafal, 2004; Paus et al., 1991; Husain, Parton, Hodgson, Mort, & Rees, 2003; Walker, Husain, Hodgson, Harrison, & Kennard, 1998).

Other work in the domain of psycho-linguistics suggests that automatic programming of saccades may also occur in response to linguistic cues. In the so-called “visual world” paradigm and its variants, participants listen to an auditory description of a required action and associated object–object/object–action relationships whilst viewing a naturalistic visual scene (Tanenhaus, Spivey-Knowlton, Eberhard, & Sedivy, 1995). The manner in which participants direct their gaze to components of the display is found to mirror the process of linguistic disambiguation occurring whilst decoding the sentence. For example, Allopenna, Magnuson, and Tanenhaus (1998) constructed displays in which distractor items could be phonologically similar or unrelated to target items in the display. Within 300 ms of onset of the relevant auditory word, eye movements were more likely to be directed to the target stimulus and phonologically related items compared to unrelated items. Other work has shown that even during passive viewing of a picture whilst hearing an acoustically presented sentence, eye movements are spontaneously directed to the relevant components of the scene (Cooper, 1974; Eberhard, Spivey-Knowlton, Sedivy, & Tanenhaus, 1995; Altmann & Kamide, 1999). This strongly suggests that linguistic stimuli cause automatic programming of saccades, although this has not been directly tested in a task for which the semantic content of word stimuli must be ignored to perform the task efficiently. Establishing the existence of such direct linkages between language and saccades would considerably simplify our understanding of the processes underlying some types of linguistic communication, such as the conversational language used by individuals cooperating in the performance of a visuo-spatial task (Tanenhaus & Brown-Schmidt, 2008).

In order to directly test whether saccade programming is subject to automatic influences by linguistic cues we devised a version of the Stroop task which required a saccadic response rather than a verbal or press button response. Each trial in the task involved the presentation of a written “cue” word at the fixation point. The word either referred to a particular location (“up”, “down”, “left”, “right”) or colour (“red”, “blue”, “green”, “yellow”). In each case the subjects’ task was to respond by looking towards one of four colour patches that matched the colour in which the word was written and to ignore the word form/identity. An effect of word form on saccade latencies and/or the direction of overt errors in the task would be consistent with a direct effect of linguistic stimuli on saccade generation similar to that found for peripheral onsets.

## 2 Materials and method

### 2.1 Participants

Ten University of Exeter students and staff participated in the study (four male) aged between 22 and 40 (*M* = 27 yrs 10 mths; *SD* = 5 yrs 4 mths). All participants had normal or corrected to normal visual acuity and normal colour vision. The study was approved by the School of Psychology ethics committee, University of Exeter.

### 2.2 Display and stimuli

**Fig. 1:**
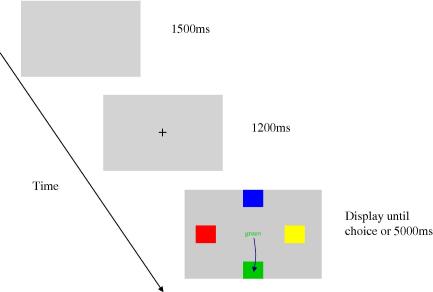
Schematic of the oculomotor Stroop task showing the sequence of events on a congruent cue trial in the Colour Word condition.

Stimuli were presented on an Iyama vision master Pro452 21 in colour monitor operating at 100 Hz. Participants were seated 60 cm from the screen and made saccadic responses towards one of four target colour “patches” in the periphery (Fig. 1). The colour patches subtended approximately 3° of arc at an eccentricity of 7.5° from the fixation point. The Stroop word stimuli were presented at fixation. In the Colour Word condition, the Stroop stimulus consisted of the word ‘red’, ‘blue’, ‘green’ or ‘yellow’ in Times New Roman font. On Congruent trials the colour words were always presented in their corresponding “ink” colour i.e. the word ‘red’ in red. For incongruent trials the colour words were displayed in an alternative colour which matched one of the peripheral response boxes i.e. the word ‘red’ displayed in blue. In the Direction Word condition the centrally presented Stroop stimulus was one of the four direction words ‘up’, ‘down’, ‘left’ and ‘right’. Congruent stimuli in this condition were composed of the direction words presented in the same colour as the patch in the corresponding location. For example the blue square was presented at the top of the screen therefore the word ‘up’ displayed in blue was congruent whereas the word ‘up’ displayed in yellow was incongruent. In both conditions Neutral trial stimuli consisted of four capital X’s presented at fixation, displayed in a colour matching one of the peripheral response location.

### 2.3 Procedure

Each condition (Colour Word/Direction Word) consisted of 108 trials run as a single block containing 36 trials each of the congruent, neutral and incongruent stimuli presented in a pseudorandom order that varied between subjects. Each congruent and neutral stimulus type was presented nine times. The order of the blocks was counterbalanced across subjects. Participants were instructed to respond to the colour of the font in which the word was presented as quickly and as accurately as possible by directing their gaze towards the colour patch which matched the font colour of the word presented in the centre of the screen. They were told that the identity of the word itself was irrelevant to the task. Instructions were displayed on the screen prior to the start of each block and the experimenter checked with each participant prior to commencing that they fully understood the task requirements. A short practice block of 10 words was presented prior to each block and excluded from the main analysis.

Each individual trial contained the following sequence of events (Fig. 1). Prior to the start of the trial a fixation point stimulus was presented centrally. This was extinguished following a period of continuous fixation lasting 1200 m. The Stroop word stimulus was then displayed at the central location simultaneously with the onset of the four peripheral colour patches. A tone sounded when participant’s gaze had been recorded at the correct target location for a period in excess of 1200 ms or at the end of a 5000 ms time out period. A blank screen was then presented for 1500 ms prior to the commencement of the next trial.

### 2.4 Eye movement recording and analysis

Eye movements were recorded using an Eyelink II Eyetracking system (SR research Ltd.), a video based pupil/CR tracker with head movement compensation system. Eye movements were sampled at 250 Hz. Calibration and validation of eye movements was carried out prior to the commencement of each trial block using a nine point calibration process.

Saccade parameters were extracted off line using Eyelink DataViewer software (SR Research Ltd). Saccades were detected using a combined velocity and acceleration criteria of 30 °/s and 8000 °/s^2^. Saccades with a latency greater than 2 standard deviations from the mean or <80 ms were excluded from analysis as were saccades with an amplitude <2°. The primary measure of interest was the latency of onset of the first saccadic response from the onset of the target colour patches and cue word. The amplitude of the initial saccade following stimulus onset was also recorded as was the angular direction of the saccade end-point relative to fixation. Saccades which deviated by ±45° from the correct target direction were classified as response errors and analysed separately.

## 3 Results

### 3.1 Analysis of correct trials

#### 3.1.1 Reaction times

**Fig. 2:**
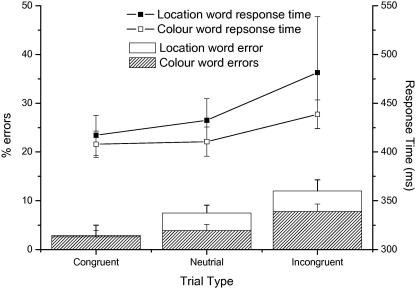
Inter-subject mean response times and error rates with standard error bars for both Colour Word and Location Word conditions.

The mean latency of response of the first saccade following onset of the word cue and response array (i.e. reaction time) were analysed across subjects using a 2 way repeated measures analysis of variance (ANOVA) with word type (Colour Word/Direction Word) and trial type (Incongruent, Congruent, Neutral) as factors. This analysis showed a significant effect of trial type (*F*(2, 18) = 5.29, *p* = 0.016), but no main effect of word type (*F*(1, 9) = 0.973). Reaction times were increased for control trials relative to trials where the word was congruent with target location, with incongruent cue trials showing the longest mean reaction times across subjects. Although mean response times indicated that the congruency effect was larger in the Colour Word condition, the interaction effect between word type and trial type did not reach significance (*F*(2, 18) = 0.43) (Fig. 2). Direct means comparisons between conditions revealed a significant difference between congruent and incongruent trial latencies (repeated measures *t*-test: *t* = 2.43, d.f. 9, *p* < 0.05) and marginally significant differences between congruent versus neutral (*t* = 1.87, *p* = 0.09) and neutral versus incongruent (*t* = 1.89, *p* = 0.09) trial latencies across subjects

### 3.2 Saccade amplitude and direction

No significant variation was found in the amplitude of the primary saccade dependent upon trial type (Congruent, Incongruent, Neutral) (*F*(2, 18) = 0.067). For incongruent trial responses classified as correct, we also examined the effect of the relative positioning of the target patch and the location congruent with the identity (rather than “ink” colour) of the cue word. Specifically we measured the angular direction of saccades relative to the central fixation point and examined how this varied dependent upon whether the “distracting” location was in a clockwise, anti-clockwise or directly opposite position relative to the correct target colour patch. This analysis revealed no significant variation in the angular deviation of the saccade trajectory dependent upon the relative location of the target and distracting location (*F*(2, 18) = 0.04).

### 3.3 Analysis of errors

On 4.6% of all trials (Congruent, Neutral and Incongruent) the first saccade following stimulus onset was executed in the wrong direction. These trials were classified as errors and analysed separately (see eye movement recording and analysis above). A 2 way analysis of variance on the number of errors made by each subject with word type (Colour Word/Direction Word) and trial type (Congruent, Neutral, Incongruent) as factors showed that error rates increased for incongruent relative to neutral and congruent trials (*F*(2, 18) = 8.23, *p* = 0.003). There was no significant main (*F*(1, 9) = 3.10, *p* = 0.11) or interaction effect of word type (*F*(2, 18) = 0.29) (Fig. 2). Direct means comparisons between conditions revealed a significant difference between congruent and incongruent (repeated measures *t*-test: *t* = 3.31, d.f. 9 *p* < 0.01) and congruent and neutral trial error rates (*t* = 4.58, *p* < 0.001), and a marginally significant differences between neutral and incongruent trial errors (*t* = 2.24, *p* = 0.05). Overall there was a trend for errors to have shorter latencies than correct responses (errors: 402 ms correct: 436 ms), but this difference did not reach statistical significance (*F*(1, 9) = 2.72, *p* = 0.13).

**Fig. 3:**
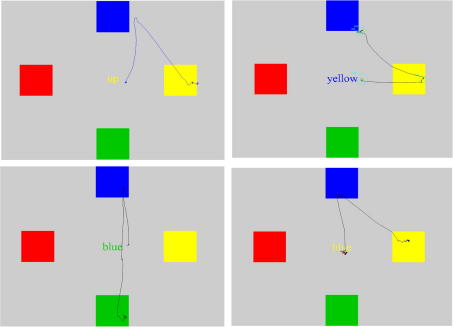
Saccade trajectory plots of error trials for which the primary saccade was directed towards the location congruent with the word cue identity rather than its “ink” colour (For interpretation of colour in Fig. 3, readers are referred to the web version of this article.)

**Fig. 4:**
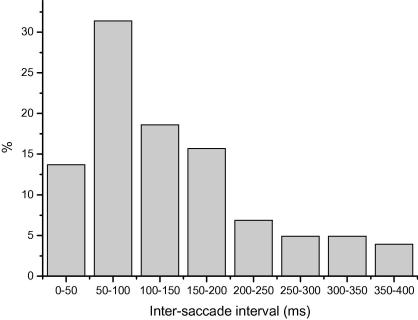
Frequency histogram for inter-saccade intervals between error saccades and subsequent corrective movements, showing percentage of corrections occurring in each 50 ms time bin following the end of the primary (errorneous) saccade.

On a given incongruent error trial the mean probability of the primary saccade being directed towards a location unrelated to the cue word was 0.17, whereas the probability of an error being directed to the location congruent with the cue word identity was 0.67 (compared to the expected probability of 0.33 for both location types). An ANOVA contrasting the probability of an error being directed towards locations which were either congruent or incongruent with the Colour/Location word conditions revealed a significant bias towards the location congruent with the word identity across subjects (*F*(1, 9) = 7.651, *p* = 0.022). Importantly, actual decision errors where subjects failed to correctly fixate the correct colour patch at the end of the trial never occurred. Saccades directed to an incorrect location were always followed by secondary movements towards the correct colour patch, indicating that all subjects correctly understood the task (see Fig. 3). The frequency distribution of inter-saccade intervals following errors is shown in Fig. 4, showing that the many errors were followed by corrective saccades within 100 ms of the end of the primary saccade. Finally, we examined the relationship between the inter-saccade interval and the amplitude of the primary saccade. Although a trend was observed towards shorter inter-saccade interval errors to be preceded by smaller amplitude saccades in some participants, this effect was not found to be consistent across individuals and was not statistically significant (Pearson correlation coefficient amplitude versus inter-saccade interval = 0.032).

## 4 Discussion

The demonstration of a saccadic Stroop effect indicates that the presentation of linguistic stimuli can influence saccade programming even when word form is irrelevant to task performance. Saccade latency was increased when the Stroop word was incongruent with the Location/Colour of the correct response location (instructed by the “ink” colour of the Stroop word) compared to congruent cue trials (Fig. 2). Further, overt saccade errors were observed towards the location matching the word form rather than text colour at a rate above that expected by chance. This suggests that written words (or more precisely the referents in their meaning) can also sometimes “capture” saccadic eye movements in a manner similar to that previously reported for peripheral onsets (e.g. Hallett & Adams, 1980; Ross & Ross, 1980; Theeuwes et al., 1998; Walker et al., 1997).

Traditionally, oculomotor researchers have made a distinction between reflexive/exogenously driven saccades towards peripheral stimuli and voluntary/endogenously driven movements instructed via symbolic cues (including written words and verbal instructions). An analogous distinction is also often made in the domain of covert attentional shifts in the absence of eye movements (Posner, 1980; Müller & Rabbitt, 1989; Crawford & Müller, 1992). However, the present experiment suggests that central/symbolic cues can influence saccade programming in a more direct/involuntary manner. Consistent with the present findings, other researchers have reported a biasing effect of symbolic cues such as arrows and numerical digits on covert attention and eye movements, even when such cues are uninformative concerning likely target locations (Fischer, Warlop, Hill, & Fias, 2004; Tipples, 2002). Other work has also revealed that short latency orienting eye movements are made to relevant objects in naturalistic visual scenes when viewers simultaneously listen to an auditory speech stream describing the scene (Altmann & Kamide, 1999).

Whilst the neural circuitry underlying the control of saccades to peripheral visual stimuli is well specified (e.g. Scudder, 1988) the influence of higher level factors is less well understood. Findlay and Walker (1999) have proposed a model of saccade programming within which separate “where” and “when” processing pathways determine the spatial end-point and onset time of saccades. The model successfully accounts for automatic capture of saccades by peripheral visual events via a direct influence of peripheral stimulus onsets on both processing pathways, but does not provide a mechanism via which symbolic cues could directly initiate saccades. The presentation of a stimulus at fixation is assumed to have an overall inhibitory effect on saccade generation within the model, with symbolic cues influencing eye movements via unspecified “cognitive” influences. We propose instead that when symbolic/linguistic stimuli have been repeatedly paired in the wider environment with peripheral stimulus onsets/saccades, direct linkages become established with associated saccade programmes. Once these learned associations have been established they are activated even in contexts where cues are unpredictive of target location/identity as in the case of the present oculomotor Stroop task.

Many other studies have used manual (key press) responses rather than the original vocal colour naming responses to index Stroop interference (MacLeod, 1991). In the manual version of the task participants learn a fixed mapping linking different colours with individual response keys (e.g. Keele, 1972; Parris, Sharma, & Weekes, 2007) and this mapping remains constant throughout the task. With this in mind we also kept the layout of the peripheral coloured response patches fixed across trials/participants in the present study. However, this aspect of the methodology leads to the possibility that our subjects learned to make direct associations between colours and corresponding spatial locations, rather than colour words and responses towards targets of the respective colour. If this were the case then randomization of the peripheral colour patch locations should lead to a reduction in the Stroop effect, particularly in the Colour Word condition. We therefore ran an additional 12 participants using an identical procedure, the only difference being that the locations at which the colour patches appeared varied randomly from trial to trial (whilst still maintaining the general up, down, left, right configuration). The results showed that the magnitude of the congruency effect on response times and error rates remained unchanged in both word type conditions (see supplementary Fig. s1 online). Further as in the main experiment erroroneous saccades on incongruent trials were far more likely to be directed to the word-congruent location than any other location in both the Direction Word and Colour Word condition (66% of incongruent trial errors were directed to the word-congruent location compared to 17% for the remaining two locations).

It is interesting to note that although errors and latencies were increased on incongruent trials in the current study, no variation was seen in the trajectory (amplitude/direction) of correct saccades on incongruent compared to congruent trials. Some studies of the manual version of the Stroop task have indicated that Stroop interference is limited to the response programming stage and is not evident in the duration or spatial characteristics of motor responses once executed (Logan & Zbrodoff, 1998). However, we cannot exclude the possibility that under some circumstances the spatial characteristics of saccades would also show effects on incongruent trials. Previous work has shown how increasing separation between target and distractor stimuli can lead to an increase in the effect of the distractor on saccade latency and a reduction in the magnitude of the effect on the spatial trajectory of saccades (McSorley et al., 2004). There appears to be a critical angular separation within which temporally contiguous saccade goals produce spatial averaging or centre-of-gravity effects on saccadic responses (Findlay, 1982; Walker et al., 1997). The coloured response patches in the present study were always orthogonal to each other, perhaps explaining why effects were only observed on errors and response latencies rather than the spatial parameters of saccades. Further research could vary the angular separation between the peripheral response locations in the saccade Stroop task to investigate this possibility.

An aspect of the current findings that has implications for more theoretical accounts of the Stroop effect is the observation of very short inter-saccade intervals between initial errors and subsequent corrective saccades (often <100 ms, i.e. shorter than a typical saccadic reaction time) (Fig. 4). Early accounts of the Stroop effect had proposed that processing of the stimulus attributes and selection of the appropriate response proceeded in a serial manner, with a processing bottleneck occurring at the response selection stage (see MacLeod for a review). However, more recent accounts have envisaged Stroop interference as the product of a parallel process in which gathering of information/evidence plays an integral role in response selection processes (Cohen, Dunbar, & McClelland, 1990; Logan, 1980). In these models a response occurs when a hypothetical output unit’s activity exceeds a threshold level. However, output units associated with other potential responses will also continue to accrue information, pushing them closer to threshold. Consistent with this idea, the very short latency corrections observed in the saccadic version of the task strongly suggest that saccadic responses are programmed in parallel to two goals defined by both the cue word identity and colour.

Future investigations could assess the extent to which damage to the frontal cerebral cortex leads to an increase in the tendency for word cues to “capture” saccadic behaviour. The saccadic Stroop task described here also has a pragmatic advantage for testing deficits in inhibitory control in neurological patients over the more commonly used manual press button response and verbal response versions of the task. This is because many patients with frontal lobe damage suffer from primary impairments in skeletomotor movements (e.g. hemiplegia) or speech production (i.e. aphasia). In contrast, sustained primary impairments in saccadic movement production following frontal lobe damage appear to be less common.

In conclusion, our results demonstrate that the presentation of linguistic cues can affect saccade programming processes even when words are irrelevant to the instructed task. We suggest that presentation of written word cues can lead to direct activation of saccadic motor programmes in a manner similar to that reported elsewhere for peripheral visual onsets.

## Figures and Tables

**Supplementary Figure S1:**
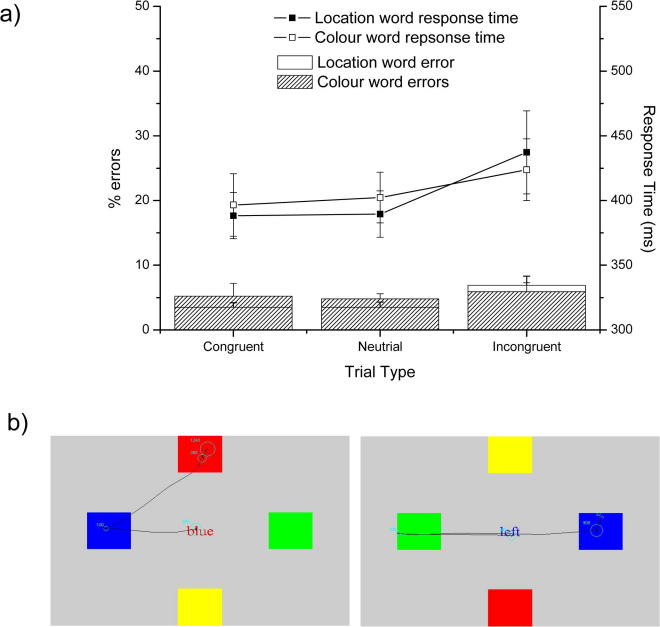
(a) Response times and error rates for control experiment in which 12 participants repeated the experiment with peripheral colour patch location randomised across trials; (b) Example “capture error” trials from the control experiment.
